# Plasma Circular RNAs in Breast Cancer: From Biomarker Potential to Functional Significance

**DOI:** 10.1002/cnr2.70316

**Published:** 2025-09-18

**Authors:** Sepideh Abdollahi, Ghasem Azizi‐Tabesh, Amirhossein Sangi Nasab Lahijan, Arman Rajabi Matak, Pantea Izadi

**Affiliations:** ^1^ Department of Medical Genetics, School of Medicine Tehran University of Medical Sciences Tehran Iran; ^2^ Genomic Research Center Shahid Beheshti University of Medical Sciences Tehran Iran

**Keywords:** biomarker, breast cancer, circular RNA

## Abstract

**Background:**

Breast cancer remains life‐threatening, but mortality declines with earlier diagnosis. Conventional work‐ups rely on invasive tissue biopsy of imaging‐detected masses. Liquid biopsy offers a minimally invasive alternative by assessing circulating biomarkers. Among these, circular RNAs (circRNAs) are compelling because their covalently closed structure confers high stability in blood. Recent studies connected circRNAs to malignancy process in breast and proposed their diagnostic potential. This review has collected relevant evidence on circRNA biogenesis, functions and their dysregulated plasma signatures in breast cancer.

**Recent Findings:**

Multiple plasma circRNAs showed diagnostic and prognostic signal in breast cancer. Upregulated hsa_circ_0001785 outperformed traditional plasma tumor markers (CEA and CA15‐3) for detection of breast cancer; higher plasma levels associated with distant metastasis, advanced TNM stage, and higher grade. Elevated hsa circ_0108942 in plasma correlated with larger tumors, lymph node involvement, and advanced stage. Hsa circ 0042881 was increased in tumors and plasma and correlated with higher TNM stage and larger tumor size. Conversely, downregulated plasma circRNAs, included hsa circ 0068033 with inverse links to stage and tumor size, and hsa_circ_0104824, both are promising for non‐invasive diagnosis of breast cancer and prognostication of breast cancer. Subtype‐specific circRNAs are also noted: circEGFR was upregulated in triple‐negative subtype and aligned with aggressive clinical features and reduced chemotherapy sensitivity, whereas circ‐FOXO3 was downregulated and associated with lymph‐node metastasis, consistent with a tumor‐suppressive role.

**Conclusion:**

Plasma circRNAs represent a biologically grounded class of minimally invasive biomarkers with promise for early detection, risk stratification, and real‐time monitoring in breast cancer. To progress toward clinical utility, priorities are larger multi‐center cohorts, harmonized reporting standards, head‐to‐head comparisons with established markers, transparent cut‐offs and prospective evaluation of multi‐marker panels integrated with imaging and clinicopathologic variables.

## 1 Introduction

As a prevalent malignancy in women all over the world, breast cancer (BC) is considered an important cause of cancer mortality. For example, 2.3 million new cases of BC were detected in women in 2020, of which 685 000 died from the disease [1]. Despite advancements in diagnostic techniques and therapeutic approaches in BC, the morbidity and death rates persist at elevated levels. However, early detection of BC has been shown to substantially reduce mortality and morbidity, as patients diagnosed at an early stage often benefit from more effective, less invasive treatments and exhibit significantly higher survival rates compared to those diagnosed at later stages. Studies indicate that early‐stage detection can improve five‐year survival rates to over 90%, underscoring the critical role of timely diagnosis in BC management [2]. Hence, early diagnosis is crucial to reduce BC mortality by an apropos treatment [3]. In this context, blood‐based biomarkers can be useful.

Blood‐based routine biomarkers such as cancer antigens (e.g., carbohydrate antigen 15‐3 (CA 15‐3) and Carcinoembryonic antigen (CEA)) used in clinical practice currently have limited sensitivity and specificity; therefore, they are not suitable enough for the BC early diagnosis. For instance, while CA 15‐3 and CEA levels may be increased in BC, they are also commonly elevated in other conditions and are typically more reliable for monitoring disease progression rather than detecting BC at an early stage [4]. Some non‐coding RNAs (ncRNAs) were discovered using conventional and cutting‐edge technologies owing to the advancements in bioinformatics and next‐generation RNA sequencing technology [5]. Recent investigations proposed several ncRNAs, including miRNAs, as biomarkers for predicting long‐term survival and early risk stratification in BC patients [6]. CircRNAs, a unique subgroup of endogenous ncRNAs, have also been evaluated due to their vital role in BC [7]. CircRNAs are single‐stranded RNAs with a covalently closed structure that are generated by a back‐splicing process. These recently identified noncoding RNAs exhibit significant tissue‐ and cell‐specific expression [8]. CircRNAs can escape from the digestion of exonuclease enzymes owing to the absence of a free 5′ or 3′ end, making them more stable and effectively expressed compared to their linear counterpart. CircRNAs can, therefore, persist in body fluids, including saliva, urine, blood, and particularly plasma [9].

The difference in plasma circRNA levels between cancer patients and healthy individuals implies that circRNAs could be crucial for the initiation and evolution of cancer. For example, researchers found a favorable correlation between the high plasma level of the hsa_circ_0007534 and adverse prognosis in colorectal cancer (CRC) [10]. Also, investigators studied the performance of circRNAs as non‐small cell lung cancer (NSCLC) biomarkers [11]. According to their findings, circ FARSA, a circRNA created through the *FARSA* gene's exon 5–7, was shown to be more prevalent in patients' plasma than controls' plasma(*p* = 0.001) and was over‐expressed in the tumor tissue (*p* = 0.016) [11]. Moreover, another research group has established the diagnostic importance of plasma circRNAs in hepatocellular carcinoma (HCC) associated with the hepatitis B virus (HBV) [12]. According to their findings, plasma hsa_circ_0027089 can be considered a novel diagnostic indicator for HCC caused by HBV [12]. A recent study has identified specific circRNAs, such as hsa_circ_0000091, hsa_circ_0067772, and hsa_circ_0000512, as promising blood‐based biomarkers for BC. These circRNAs exhibit differential expression in BC patients' plasma compared to healthy individuals, offering high sensitivity and specificity in diagnostic models. For instance, hsa_circ_0000091 has been shown to correlate with axillary lymph node metastasis, TNM stage, and overall prognosis, suggesting its utility not only in diagnosis but also as a prognostic marker. When combined with ultrasound, hsa_circ_0000091 improves predictive accuracy for lymph node metastasis, providing a minimally invasive approach to assess BC progression and aiding in tailored patient management. These findings highlight the potential of circRNAs in advancing liquid biopsy techniques for early and accurate BC detection [13]. Although, an investigation presented some circRNAs as potential blood‐based biomarkers in BC [14], to date, there has been limited experimental evidence about the significance of these circRNAs as diagnostic biomarkers and their functional role in BC. Hence, in this review, upregulated and downregulated plasma circRNAs in BC were considered as the main focus and their clinical implications were discussed. Their unique expression patterns in the plasma provide an opportunity for development of a non‐invasive diagnostic biomarker that may also be useful as a prognostic or predictive biomarker. Thus, understanding the clinical relevance of these circRNAs can pave the way for better diagnostic, prognostic, and therapeutic strategies, ultimately advancing personalized care in BC management.

## 2 CircRNAs

The early history of circRNAs dates back to the mid‐1970s when they were initially discovered in pathogens, with Sanger et al. identifying them as covalently closed, single‐stranded RNA molecules in viroids. These early observations hinted at potential biological relevance, but circRNAs were largely overlooked due to the prevailing focus on linear RNAs and RNA splicing. This initial oversight delayed the recognition of circRNAs' functional roles, which only began to gain attention with advancements in RNA sequencing technologies. Today, circRNAs are known to play critical regulatory roles across various biological processes [15]. For example, they often act as miRNA sponges, as seen with circCD44 in triple‐negative breast cancer, where it promotes tumorigenesis by binding miR‐502‐5p. Moreover, specific circRNAs have demonstrated clinical potential as non‐invasive biomarkers. In BC, circulating circRNAs (ccircRNAs) like hsa_circ_0001785 and hsa_circ_0108942 are linked to tumor progression and prognosis, reinforcing the potential of circRNAs in diagnostics and as therapeutic targets [16, 17]. This historical and functional evolution emphasizes the growing relevance of circRNAs in understanding disease mechanisms and developing clinical applications.

## 3 Biogenesis of circRNAs

**FIGURE 1:**
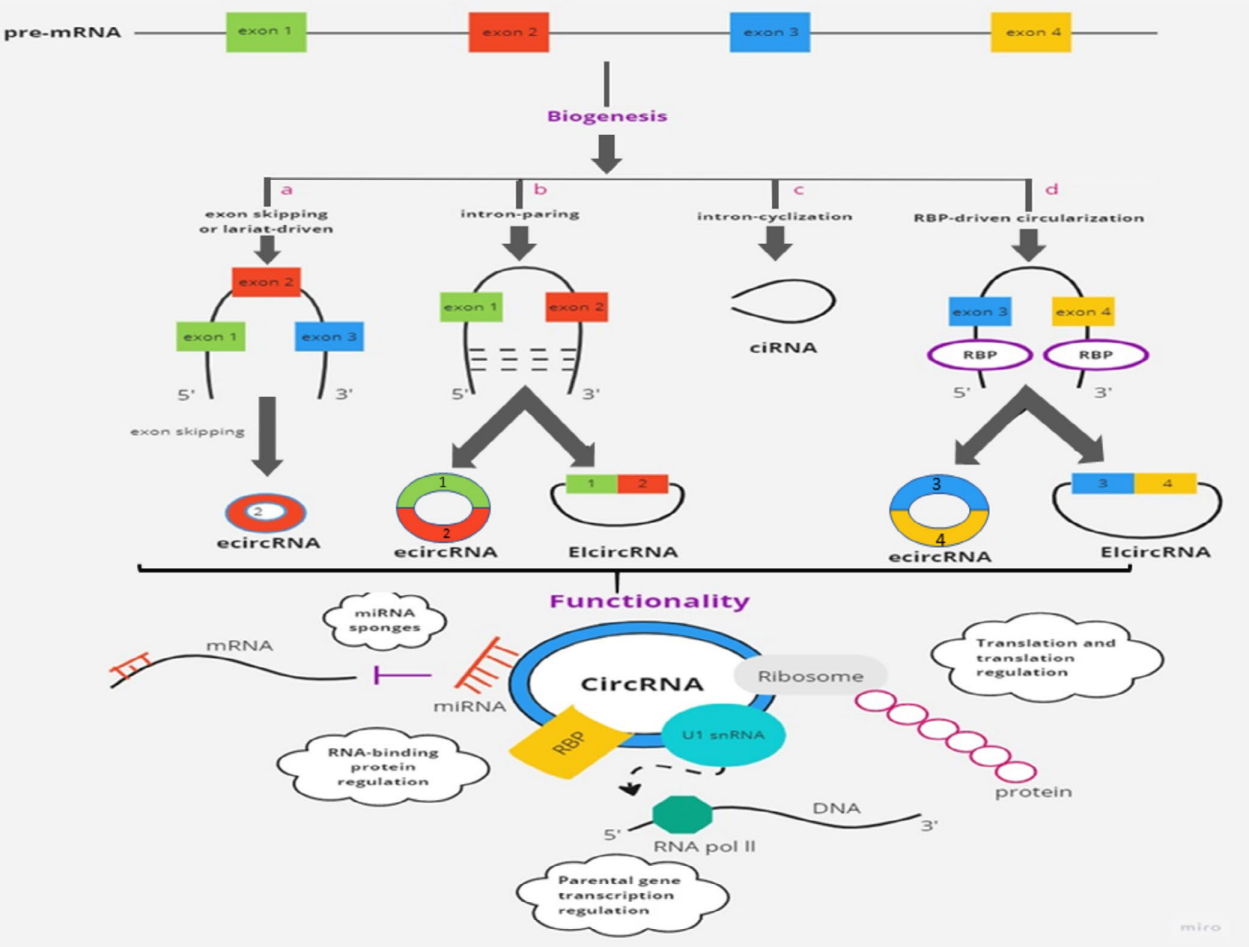
Overview of the circular RNA's synthesis and function. (a) Lariat‐based circularization or exon skipping. In this scenario, a lariat separates exon 2 by an exon‐skipping event. The intronic region is removed and the RNA becomes circularized as a result of internal splicing of this lariat. (b) Direct back‐spacing or intron pairing‐driven circularization. The adjacent introns of exons 1 and 2 include reverse repeated or corresponding sequences that pair to each other and generate a circular shape. To create EcirRNA or EIciRNA, the introns are either deleted or maintained. (c) Model of intron cyclization. A circular structure formed by an intron may be encouraged by specific conserved parts at both ends of the intron. In this approach steady ciRNAs are produced using a debranching enzyme to halt intron debranching. (d) Circularization prompted by RBP. RBPs bind to the upstream of the exons 3 and 4 and downstream introns in this instance. Then, the RBPs create a link between the introns. As a result, the 5′‐phosphate group of downstream intron binds to the upstream 2′‐hydroxyl group. circRNA: Circular RNA; EcirRNA: Exonic circRNA; EIciRNA: Exon‐intron circRNA; RBP: RNA‐binding protein.

There are three groups of circRNAs in terms of their structural characteristics: (1) Circular intronic RNAs (ciRNAs), which are made up entirely of introns; (2) Exon‐intronic RNAs (ElciRNAs), which are composed of introns and exons and have interaction with U1 snRNP to control gene transcription; and (3) Exonic circRNAs (EcircRNAs), which are made entirely of exons [18]. In terms of circRNAs biogenesis, some models were proposed [19]:

- Lariat‐based circularization or exon skipping which the lariat shape was created when the RNA was folded. It has been shown that rapid splicing produces circRNAs and eliminates intron sequences in the lariat species [19].
- Intron‐pairing‐driven circularization or direct back‐splicing: At both ends of a pre‐mRNA, there are complementary sequences in pairs or inverted repeats that are part of the flanking introns. In this process, EcirRNA or EIciRNA are created by either deleting or maintaining the introns [20].
- Intron cyclization model: After splicing, a circular structure can be created from introns by preventing debranching enzyme activity, which can lead to intron debranching. As a result, stable circRNA is generated due to the conserved properties in both ends of the intron [21, 22].
- RBP‐driven circularization: RNA‐binding proteins like ADAR (adenosine deaminase acting on RNA) and QKI (Quaking) are crucial modulators of circRNA synthesis, influencing the circularization of RNA by interacting with specific intronic sequences near circRNA‐forming exons. ADAR edits double‐stranded RNA by converting adenosine to inosine (A‐to‐I editing), which alters the stability of RNA duplexes that typically form near splice sites. This editing disrupts base pairing in introns that flank circRNA regions, thereby reducing circRNA production. Studies have shown that ADAR knockdown can lead to increased circRNA levels, suggesting that ADAR antagonizes circRNA formation by destabilizing the necessary RNA structures for circularization. On the other hand, QKI actively promotes circRNA formation by binding to QKI response elements in introns flanking circRNA‐forming exons. During processes like epithelial‐to‐mesenchymal transition (EMT), QKI levels increase, leading to enhanced circRNA production. By bringing exons into closer proximity, QKI facilitates the back‐splicing needed to form circRNAs. Experimental insertion of QKI binding motifs has been shown to induce circRNA formation even in transcripts that typically undergo linear splicing, further underscoring its role in circularization [23, 24]. (Figure 1)

## 4 Functions of circRNAs

Generally, because of their distinctive structural characteristics, circular RNAs are significantly more durable than linear RNAs and resistant to RNA exonuclease breakdown [25]. They are broadly expressed in the body in a tissue‐specific manner. CircRNAs make up approximately 2%–4% of all mRNA transcripts, although some are particularly prevalent in specific cell types like fibroblasts [26]. It has been indicated that circRNAs can target RNAs and proteins either directly or indirectly, thereby controlling gene expression at several levels as a result (Figure 1). Their regulatory functions include:

### 4.1 miRNA sponge

miRNAs are endogenous small RNAs (length 22 nucleotides) that target the 3'UTRs end of mRNAs and regulate their translation. The activity of miRNAs is influenced by miRNA sponges, which is done by circRNAs as competitive endogenous RNAs (ceRNAs) [27]. Thus, they can sequester miRNAs and reduce their access to their target mRNAs. This action disrupts the normal miRNA regulatory function, which can affect the expression level of their mRNA targets indirectly [28]. This concept is exemplified by the circular CDR1, which acts as a sponge for miR‐7. Circular CDR1 contains multiple miR‐7 binding sites, effectively sequestering miR‐7 and preventing it from suppressing its target mRNAs such as insulin‐like growth factor 1 (IGF1), insulin receptor substrate 2 (IRS2) and epidermal growth factor receptor (EGFR), thereby influencing cell survival through signaling pathways such as PI3K/AKT, NF‐kappaB, and the epithelial‐mesenchymal transition (EMT) [29, 30].

### 4.2 RNA‐binding protein regulation

CircRNAs can interact with proteins, thus affecting the activity of target proteins while serving as miRNA sponges [31]. To interact with RBP, circRNA has to bind to specific locations. This mechanism is bidirectional and can affect circRNA production and degradation in addition to protein expression and gene transcription [31]. One such example is circ‐Foxo3, which affects the survival of cells through interactions with p21 and CDK2 [32]. Another instance is the circular RNA circ‐Ccnb1, which is originated from exon 4 and exon 5 of the *Cyclin B1* gene and interacts with Bclaf1 and H2AX in p53 altered cells to create a complex that causes BC cells to perish. However, in p53 wild‐type cells, through its binding to H2AX, circ‐Ccnb1 can impair the mechanisms that reduce carcinogenesis [33].

### 4.3 Transcription regulation

circRNAs may take a part in controlling the expression of their parent genes. Certain circRNAs may interact with transcription factors or RNA polymerase II in the cell nucleus. For example, EIciRNAs such as circEIF3J and circPAIP2 can interact with U1 small nuclear ribonucleoprotein (snRNP) through RNA–RNA interaction. Then these EIciRNA‐U1 snRNP complexes interact with the transcriptional complex, which exists in the parental gene promoter and can enhance gene expression [34].

### 4.4 Translation regulation

Translation of some circRNAs is possible by ribosomes, despite evidence showing that the majority of them cannot produce proteins [35]. This capability arises from specific structural features such as internal ribosome entry sites (IRESs), which facilitate cap‐independent translation, and N6‐methyladenosine (m6A) modifications that enhance translation initiation. For instance, circRNAs like circ‐ZNF609, circ‐SHPRH, and circ‐FBXW7 are translated into functional proteins and contain open reading frames (ORFs) with sequences allowing efficient translation. These proteins play pivotal roles in cellular functions; for example, circ‐SHPRH generates a tumor‐suppressive protein (SHPRH‐146aa), while circ‐FBXW7 produces FBXW7‐185aa, which suppresses glioma tumorigenesis [36, 37]. In addition to encoding proteins, circRNAs can regulate the translation of linear mRNAs. For instance, circPABPN1 competes with PABPN1 mRNA for binding to human antigen R (HuR), a protein essential for promoting PABPN1 translation. By sequestering HuR, circPABPN1 reduces its availability for the mRNA, resulting in decreased translation of PABPN1 [38]. This represents a novel regulatory mechanism where a circRNA modulates translation by competing with its linear counterpart for RBPs.

## 5 Identification Techniques of circRNAs

Due to the potential biomarker capacity of plasma circRNAs, transcriptomic techniques such as microarray or RNA sequencing (RNA‐Seq) are the main methods for discovering circRNAs expression profiles in BC [39].

### 5.1 Microarray Technology

The technical process for investigating circRNAs via microarray is similar to that for linear mRNAs, except for probe design. Probes specifically target the unique'back‐splice junctio' (BSJ) site within the circRNA sequence. By profiling these expression patterns, differentially expressed circRNAs can be identified and selected as candidate biomarkers for further analysis. However, microarrays are limited by their reliance on pre‐designed probes, which may overlook novel circRNAs [40].

### 5.2 RNA Sequencing (RNA‐Seq)

RNA‐Seq allows for objective profiling and in‐depth transcriptome analysis without requiring species‐ or transcript‐specific probes. It can identify alterations that hybridization‐based methods like microarrays cannot detect. RNA‐Seq offers a broader dynamic range for expression evaluation and greater sensitivity for low‐expression genes, which are particularly important in early cancer detection. Additionally, RNA‐Seq workflows often include RNase R treatment to degrade linear RNA, enriching the sample for circRNAs. Bioinformatics tools like CIRCexplorer, find_circ, and CIRI are critical for analyzing RNA‐Seq data, identifying BSJs, and annotating circRNA candidates [41].

After identifying promising circRNAs through transcriptomic methods, validation using target‐specific techniques is necessary to confirm their diagnostic potential:

### 5.3 Quantitative Reverse Transcription PCR (qRT‐PCR)

Widely used for validating circRNA expression, qRT‐PCR employs divergent primers designed to amplify BSJs specifically. This method is reliable and widely adopted but may be limited in sensitivity for low‐abundance circRNAs [42].

### 5.4 Reverse Transcription‐Droplet Digital PCR (RT‐ddPCR)

RT‐ddPCR is a third‐generation PCR technique that offers higher sensitivity and precision than qRT‐PCR. By partitioning samples into thousands of droplets, it enables absolute quantification of circRNAs even at very low concentrations [43]. This makes RT‐ddPCR particularly valuable for detecting low‐abundance circRNAs, a crucial feature for early BC diagnosis [44].

### 5.5 Reverse Transcription‐Rolling Circle Amplification (RT‐RCA)

This method amplifies circRNAs using ProtoScript II reverse transcriptase with a DNA primer, creating repetitive sequences of the circular RNA template. Fluorescent molecular beacons are then added, emitting strong signals when the amplified product opens its hairpin structures. RT‐RCA is highly specific to circRNAs, distinguishing them from linear RNAs with the same sequence. It is cost‐effective, sensitive, and suitable for large‐scale applications [45].

### 5.6 Loop‐Mediated Isothermal Amplification (LAMP)

LAMP is a rapid and sensitive method for circRNA detection that operates at a constant temperature (typically 65°C). This technique uses a set of primers targeting BSJs to amplify circRNAs efficiently in plasma samples. Due to its simplicity, speed, and ability to function without thermal cycling, LAMP is particularly suited for point‐of‐care testing. It is emerging as a valuable tool in resource‐limited settings and for real‐time monitoring of plasma circRNAs as diagnostic biomarkers [46, 47, 48, 49].

These advanced methodologies collectively enhance the detection, quantification, and functional analysis of circRNAs in clinical samples. By integrating high‐throughput discovery with targeted validation, researchers can reliably identify plasma circRNAs as diagnostic and prognostic biomarkers in breast cancer. Furthermore, the development of rapid and cost‐effective techniques like RT‐ddPCR and LAMP holds promise for future applications in early cancer detection, personalized treatment monitoring, and point‐of‐care diagnostics.

## 6 Liquid Biopsy in BC Based on the Plasma circRNAs Expression

A liquid biopsy is a non‐invasive diagnostic approach that involves analyzing non‐solid biological fluids, such as blood, saliva, and urine, to detect and monitor various disease states, including cancers. Unlike traditional tissue biopsies, which require invasive procedures, liquid biopsies provide a minimally invasive method for ongoing disease surveillance and early detection. Among the components analyzed in liquid biopsies, non‐coding RNAs (ncRNAs) have gained considerable attention as potential biomarkers in cancer diagnostics. Previously dismissed as “junk” due to their non‐coding nature, ncRNAs are now recognized for their roles in gene regulation and cancer pathogenesis. Types of ncRNAs, including microRNAs (miRNAs) and long non‐coding RNAs (lncRNAs), exhibit dysregulated expression patterns in various cancers, making them suitable candidates for cancer detection and monitoring.

**FIGURE 2:**
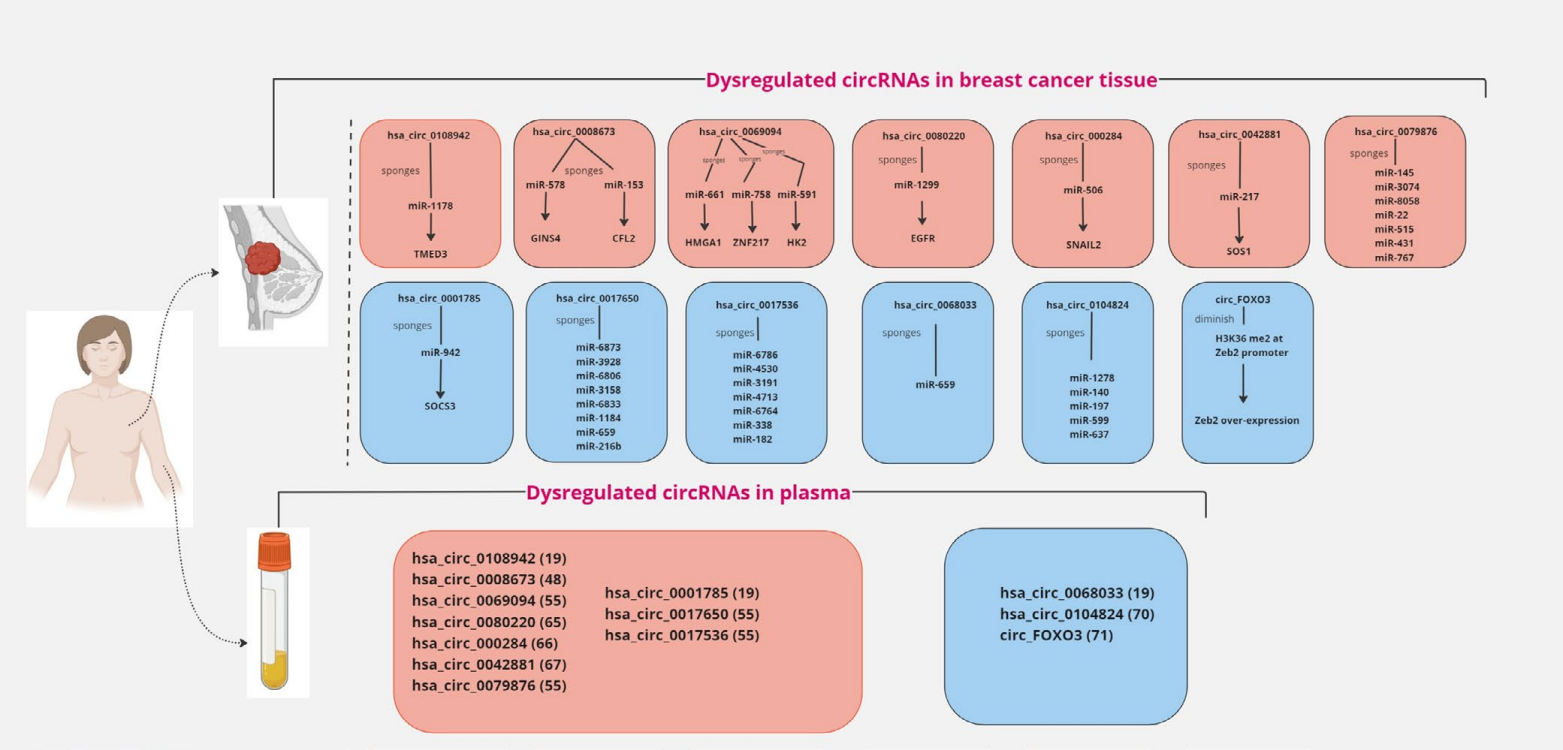
Overview of circRNAs dysregulation and their molecular interactions in breast cancer pathogenesis. This diagram illustrates dysregulated circular RNAs (circRNAs) in breast cancer tissues and plasma. These circRNAs function primarily as miRNA sponges, impacting the expression of key oncogenes and tumor‐suppressor genes. The figure highlights how specific circRNAs contribute to breast cancer progression by regulating gene expression, cell proliferation, migration, and other critical processes involved in tumor metastasis. CircRNAs upregulated in breast cancer are indicated in red boxes, while downregulated circRNAs are shown in blue boxes. Key target genes include SOCS3 (Suppressor of Cytokine Signaling 3), TMED3 (Transmembrane P24 Trafficking Protein 3), CFL2 (Cofilin 2), GINS4 (GINS Complex Subunit 4), HMGA1 (High Mobility Group AT‐Hook 1), ZNF217 (Zinc Finger Protein 217), HK2 (Hexokinase 2), EGFR (Epidermal Growth Factor Receptor), SNAIL2 (Snail Family Transcriptional Repressor 2), SOS1 (SOS Ras/Rac Guanine Nucleotide Exchange Factor 1), and Zeb2 (Zinc Finger E‐Box Binding Homeobox 2). This figure provides a visual summary of the molecular roles of dysregulated circRNAs in breast cancer, offering insights into potential mechanisms for targeted therapies and diagnostic biomarkers.

CircRNAs class of ncRNAs, possess characteristics such as sequence conservation, stability, and tissue specificity, making them promising candidates as non‐invasive diagnostic biomarkers for BC. These circRNAs display distinct expression patterns in BC compared to normal samples, and several studies have highlighted specific plasma circRNAs as promising diagnostic biomarkers in BC [17, 50]. Furthermore, due to their covalently closed circular structure, circRNAs are resistant to exonuclease degradation and remain stable in bodily fluids, including blood, saliva, and urine. This stability enhances their detectability in non‐invasive liquid biopsies. CircRNAs also exhibit cell‐ and tissue‐specific expression and play key regulatory roles in cancer‐related processes, such as serving as miRNA sponges. For instance, specific circRNAs in BC can sequester oncogenic or tumor‐suppressive miRNAs, influencing pathways crucial for tumor development and progression. These properties, coupled with their ability to reflect real‐time disease dynamics through circulating circRNA levels, underscore their potential as powerful diagnostic and prognostic biomarkers, particularly for monitoring tumor progression and treatment response in BC [33, 51, 52]. A deeper understanding of circRNAs' roles in the pathophysiology of BC and their expression profiles in plasma can pave the way for their clinical application as both diagnostic and therapeutic tools. Hence, we have discussed several recently identified upregulated and downregulated plasma circRNAs in BC patients, with an emphasis on their potential impact on BC pathogenesis (Figure 2) (Tables 1, 2).

**TABLE 1:** Upregulated plasma circRNAs in breast cancer.

CircRNA/Host Gene	Potential clinical utility	AUC	Clinicopathological associations	Expression concordance between plasma & tumors/cell lines	Pathways
hsa_circ_0108942/ANKRD12 [17]	Diagnostic, prognostic	0.70	Tumor growth and cell migration	NR	Wnt/β‐catenin [53]
hsa_circ_0008673/BRCA1 [54]	Diagnostic, prognostic, predictive	0.83	Larger tumor size, metastasis, and ER/PR positive	Cell lines (MCF‐10A, MDA‐MB‐231, MCF‐7)	DNA replication, cell motility [55]
hsa_circ_0069094/S100P [56]	Diagnostic, prognostic	0.68	Metastasis and higher metabolism	Tumors and cell lines (MCF‐7, MDA‐MB‐453, MDA‐MB‐231, MDA‐MB‐468, Hs‐578 T)	EMT, glycolysis [57]
hsa_circ_0001785/ELP3 [17]	Diagnostic, prognostic	0.78	Histological grade, TNM stage, distant metastasis	NR	JAK/STAT [58]
hsa_circ_0080220/EGFR [59]	Prognostic	NR	Chemotherapy resistance	Tumors and cell line (MDA‐MB‐231)	EGFR [59]
hsa_circ_000284/SNAIL2 [60]	Diagnostic	NR	TNM stage and lymph node involvement	Tumors	EMT [60]
hsa_circ_0042881/NF1 [61]	Diagnostic, prognostic	0.80	Larger tumor size and TNM stage	Tumors	RAS/MEK/ERK, PI3K/AKT [61]
hsa_circ_0079876/ANLN [56]	Diagnostic	0.62	TNM stage, lymph node infiltration, and Ki67	Tumors	Unknown
hsa_circ_0017650/ITIH5 [56]	Diagnostic	0.75	TNM stage, lymph node infiltration, and Ki67	Only in plasma	Unknown
hsa_circ_0017536/AKR1C1 [56]	Diagnostic	0.61	TNM stage, lymph node infiltration, and Ki67	Only in plasma	Unknown

Abbreviations: AUC, area under a curve; CFL2, cofilin 2; EGFR, epidermal growth factor receptor; EMT, epithelial‐mesenchymal transition; ER, estrogen receptor; GINS4, GINS complex subunit 4; HK2, hexokinase 2; HMGA1, high mobility group AT‐hook 1; NR, not‐reported; PR, progesterone receptor; SNAIL2, snail family transcriptional repressor 2; SOCS3, suppressor of cytokine signaling 3; SOS1, SOS Ras/Rac guanine nucleotide exchange factor 1; TMED3, Transmembrane p24 trafficking protein 3; TNBC, triple‐negative breast cancer; ZNF217, zinc finger protein 217.

**TABLE 2:** Downregulated plasma circRNAs in breast cancer.

CircRNA/Host gene	Potential clinical utility	AUC	Clinicopathological associations	Expression concordance between plasma & tumors/cell lines	Pathways
hsa_circ_0068033/NAALADL2 [62]	Diagnostic, prognostic	0.84	Tumor size, TNM stage	Cell lines (MCF‐7, MDA‐MB‐231)	Apoptosis [62]
hsa_circ_0104824/NTRK3 [63]	Diagnostic	0.82	Tumor sizes, ER positive, PR positive	Tumors	Unknown
circ_FOXO3/FOXO3 [64]	Prognostic	NR	Lymph node metastasis	Tumors	EMT [64]

Abbreviations: AUC, area under a curve; BC, breast cancer; EMT, epithelial‐mesenchymal transition; ER, estrogen receptor; NR, not‐reported; PR, progesterone receptor; TNBC, triple‐negative breast cancer; WHSC1, Wolf‐Hirschhorn syndrome candidate 1; Zeb2, zinc finger e‐box binding homeobox 2.

## 7 Upregulated Plasma circRNAs in BC Patients

### 7.1 Hsa_circ_0001785

Hsa_circ_0001785 has been investigated for its potential as a biomarker in BC. Researchers conducted a microarray analysis of plasma samples from BC patients, identifying 41 dysregulated circRNAs, including the significant upregulation of hsa_circ_0001785 compared to normal controls [17]. Validation using RT‐PCR confirmed the marked increase of hsa_circ_0001785 in BC patient plasma. To evaluate its diagnostic value, a receiver operating characteristic (ROC) curve analysis was performed, revealing an area under the curve (AUC) value of 0.78 for hsa_circ_0001785 (sensitivity = 0.76, specificity = 0.69). This AUC was notably higher than commonly used BC biomarkers such as CEA (AUC = 0.56, sensitivity = 0.43, specificity = 0.51) and CA 15‐3 (AUC = 0.62, sensitivity = 0.50, specificity = 0.57), indicating hsa_circ_000178's strong potential as a non‐invasive diagnostic tool for BC [65].

Further analysis demonstrated a significant correlation between hsa_circ_0001785 expression levels and clinical features of BC. Increased levels of this circRNA were positively associated with distant metastasis (*p* = 0.016), advanced tumor‐node‐metastasis (TNM) stages (*p* = 0.008), and higher histological grades (*p* = 0.013), highlighting its relevance in assessing disease severity and progression in BC patients [17].

In vitro and in vivo studies have explored the mechanistic role of hsa_circ_0001785 in BC pathophysiology. Interestingly, while hsa_circ_0001785 is highly expressed in the plasma of BC patients, its levels are comparatively low in BC tissue, suggesting a complex regulatory mechanism [58]. Functionally, hsa_circ_0001785 acts as a miRNA sponge, specifically binding to miR‐942. By sequestering miR‐942, hsa_circ_0001785 indirectly upregulates its target gene, suppressor of cytokine signaling 3 (SOCS3), a known regulator in the JAK/STAT signaling pathway. SOCS3 inhibits JAK/STAT signaling, which plays a critical role in cellular growth and differentiation processes, and its upregulation through hsa_circ_0001785 reduces cancer cell proliferation, invasion, and migration. This interaction highlights hsa_circ_000178's potential role in regulating signaling pathways that are fundamental to BC cell survival and metastasis [58]. This finding confirms previous results which showed that, in BC cells, over‐expression of the SOCS3 gene can lead to reduced proliferation [66]. However, the low level of hsa_circ_0001785 expression in BC cells along with the overexpression of this circRNA in plasma needs more investigations. These findings collectively suggest a complex role of hsa_circ_0001785 in BC pathophysiology, highlighting its potential as a target for therapeutic intervention and its importance in understanding BC progression.

### 7.2 Hsa_circ_0108942

Hsa_circ_0108942 has been identified as significantly upregulated in the plasma of BC patients, with an AUC of 0.70, indicating its potential as a non‐invasive biomarker for BC diagnosis [17].

In addition to its diagnostic value, hsa_circ_0108942 expression levels are associated with clinical parameters indicative of disease progression. Higher levels of hsa_circ_0108942 in plasma correlate with advanced stages of BC, increased tumor size, and lymph node involvement, suggesting its relevance in evaluating disease severity and metastatic potential. These associations reinforce the utility of hsa_circ_0108942 in monitoring BC progression and patient prognosis [17].

The molecular mechanisms of hsa_circ_0108942 in BC pathogenesis have been investigated in vivo [67]. This circRNA acts as a miRNA sponge, specifically binding to miR‐1178‐3p, which leads to the upregulation of transmembrane p24 trafficking protein 3 (TMED3). TMED3 overexpression has been previously linked to tumor growth in malignancies such as hepatocellular carcinoma [53]. In BC, TMED3 plays a crucial role in activating the Wnt/β‐catenin signaling pathway, which is known to promote cell migration, proliferation, and invasion [68]. This signaling pathway is essential in BC progression, as it influences pathways involved in tumor metastasis and cellular survival [67]. This comprehensive analysis underscores the complex interplay of hsa_circ_0108942 with BC progression and its significant implications for future therapeutic strategies and diagnostic purposes.

### 7.3 Hsa_circ_0008673

Hsa_circ_0008673 has been identified as significantly upregulated in the plasma of BC patients, showing a strong diagnostic value (AUC = 0.83). This circRNA is associated with advanced clinical features, including larger tumor size, distant metastasis, and positive estrogen receptor (ER) and progesterone receptor (PR) status, underscoring its relevance in assessing disease progression and hormonal receptor status in BC [54].

The expression of hsa_circ_0008673 correlates with clinical parameters indicative of aggressive BC phenotypes, making it a useful biomarker for disease severity and potential metastatic spread. Its association with ER and PR positivity also suggests a role in hormonal receptor signaling, which is crucial for guiding BC treatment [54].

This result is in line with the previous research, which assessed the hsa_circ_0008673 molecular mechanism in BC pathogenesis [69]. At the molecular level, hsa_circ_0008673 influences cancer progression through key signaling pathways. By sponging miR‐578, it upregulates GINS4, a protein integral to DNA replication and cell cycle progression. The miR‐578/GINS4 axis promotes cell proliferation and migration, contributing to BC pathogenesis [69]. GINS4 has been previously associated with oncogenic activities in BC, functioning within the DNA helicase complex to support rapid cell division, which is a hallmark of cancer progression [70]. Furthermore, hsa_circ_0008673 engages in another signaling axis involving miR‐153‐3p and Cofilin 2 (CFL2), an actin‐binding protein essential for cytoskeletal rearrangement and cell motility [55]. In BC cells, hsa_circ_0008673 upregulates CFL2 by sponging miR‐153‐3p, thereby promoting migration and invasion. CFL's role in regulating actin dynamics is well‐established in cancer metastasis, where it facilitates cellular movement and invasive potential [55]. Additionally, this accords with earlier studies, which showed the role of CFL2, an actin‐binding protein, in the proliferation and migration of some cancers such as gastric cancer [71], prostate cancer [72] and BC [73, 74]. These insights facilitate a deeper understanding of its clinical significance and potential utility in developing targeted cancer therapies.

### 7.4 Hsa_circ_0069094

Hsa_circ_0069094 has been identified as significantly upregulated in the plasma of BC patients, showing diagnostic value with an AUC of 0.68 [56]. Additionally, BC cell lines exhibit elevated levels of hsa_circ_0069094 compared to normal cell lines. Researchers have identified S100P as the host gene (HG) for hsa_circ_0069094 [56]. S100P encodes S100 calcium‐binding protein P, a protein involved in cell cycle progression and differentiation. High expression levels of S100P have been linked to reduced survival rates in BC patients and may facilitate metastasis [75, 76].

The elevated levels of hsa_circ_0069094 in both BC plasma and tissue samples correlate with more advanced disease characteristics, suggesting that this circRNA could serve as a biomarker for aggressive BC phenotypes [56].

In addition to the interaction of hsa_circ_0069094 with S100P, some miRNAs such as miR‐495‐3p, miR‐661, miR‐542‐3p, miR‐4504, miR‐6837‐5p, and miR‐5092 were added as targeted miRNAs for hsa_circ_0069094 by researchers using circRNA‐miRNA interaction analysis [56]. In line with their results, it has been reported that miRNA‐661 is one of the targets of hsa_circ_0069094 [77]. In BC tissue and cells, elevated levels of hsa_circ_0069094 sponge miR‐661, resulting in the overexpression of high mobility group A1 (HMGA1), a protein that promotes cell survival and proliferation [77]. HMGA1 has been implicated in the initiation and progression of BC through its influence on cancer cell survival and proliferative mechanisms [78]. Another pathway associated with hsa_circ_0069094 involves the miR‐758‐3p/ZNF217 axis. Hsa_circ_0069094 regulates ZNF217, an oncogene linked to BC metastasis, by sponging miR‐758‐3p. ZNF217 overexpression contributes to the epithelial‐mesenchymal transition (EMT), which is associated with poor prognosis and metastatic potential in BC patients [79]. This mechanism aligns with previous findings demonstrating the role of ZNF217 in promoting EMT and tumor invasiveness [80, 81]. Additionally, the miR‐591/HK2 axis represents another signaling pathway influenced by hsa_circ_0069094. In this pathway, hsa_circ_0069094 sponges miR‐591, leading to the upregulation of hexokinase 2 (HK2), a key enzyme in glycolysis [57]. The overexpression of HK2 supports the high energy demands of rapidly proliferating BC cells by enhancing glycolytic activity. This metabolic adaptation, driven by hsa_circ_0069094, aligns with prior research showing the critical role of HK2 in cancer cell metabolism and the reliance of malignant cells on glycolysis to sustain rapid growth [82]. This association with BC malignancy underscores the metabolic adaptations driven by hsa_circ_0069094, echoing prior findings on the role of HK2 in cancer cell metabolism.

### 7.5 Hsa_circ_0079876, Hsa_circ_0017650, Hsa_circ_0017536

Hsa_circ_0079876 is significantly upregulated in the plasma of BC patients, with an AUC of 0.62, suggesting its potential as a diagnostic marker for BC [56]. Additionally, hsa_circ_0079876 is also overexpressed in BC tissue, further indicating its relevance in the disease. Predictive analyses have identified several miRNAs that hsa_circ_0079876 may target, including miR‐3074‐5p, miR‐8058/5009‐5p, miR‐22‐5p, miR‐145‐5p, miR‐515‐5p, miR‐431‐5p, and miR‐767‐3p [56]. These miRNA interactions suggest a complex regulatory network, as hsa_circ_0079876 may act by sponging multiple miRNAs involved in cancer‐related pathway association with clinical parameters [56]. The expression profiles of hsa_circ_0079876, along with hsa_circ_0017650 and hsa_circ_0017536, indicate their association with disease characteristics.

Elevated levels of these circRNAs in plasma may correlate with disease progression, while the paradoxically lower expression of hsa_circ_0017650 and hsa_circ_0017536 in BC tissue suggests a complex expression dynamic that warrants further exploration. Such differences may offer insights into the transport or release mechanisms of circRNAs from tumor tissue into circulation, which could provide additional markers for disease monitoring and assessment of BC aggressiveness [56].

Hsa_circ_0079876 is predicted to interact with several miRNAs implicated in cellular processes relevant to cancer. Through miRNA sponging, it may regulate pathways that influence cell proliferation, migration, and apoptosis in BC. Similarly, hsa_circ_0017650 and hsa_circ_0017536 are thought to target a unique set of miRNAs, with hsa_circ_0017650 potentially interacting with miR‐6873‐3p, miR‐3928‐5p/6806‐3p, miR‐3158‐5p, miR‐6833‐3p, miR‐1184, and miR‐659, and hsa_circ_0017536 predicted to sponge miR‐6786‐5p, miR‐4530, miR‐3191‐5p, miR‐4713‐3p, miR‐6764‐3p, and miR‐338‐3p. These interactions suggest that each circRNA may contribute to the modulation of cancer‐related signaling pathways, potentially impacting BC cell survival, migration, and tumor progression [56]. A deeper investigation into these circRNAs' roles and interactions could offer valuable insights into targeting specific molecular pathways to inhibit BC progression.

### 7.6 Hsa_circ_0080220

Hsa_circ_0080220, also known as circEGFR, is significantly upregulated in triple‐negative breast cancer (TNBC) cell lines, patient tissues, and plasma exosomes [59]. This elevated expression level is associated with poor prognosis and suggests potential utility as a diagnostic marker for distinguishing TNBC from normal breast tissue, associating with clinical parameters [59].

The overexpression of circEGFR is linked to more aggressive clinical features in TNBC, including enhanced proliferation, migration, invasion, and epithelial‐mesenchymal transition (EMT). These characteristics align with the highly invasive and metastatic nature of TNBC, making circEGFR a valuable marker for assessing disease severity and progression [59].

CircEGFR exerts its oncogenic effects primarily through the circEGFR/miR‐1299/EGFR signaling pathway. By sponging miR‐1299, circEGFR modulates the expression of epidermal growth factor receptor (EGFR), a critical driver in TNBC progression. EGFR signaling is well‐established in promoting cell proliferation, survival, and EMT, which are central to TNBC pathophysiology. The circEGFR/miR‐1299/EGFR axis thus represents a key regulatory pathway by which circEGFR contributes to TNBC's aggressive behavior. Notably, circEGFR overexpression is also associated with reduced sensitivity to the chemotherapy drug pirarubicin, complicating treatment strategies for TNBC. In vivo studies have demonstrated that silencing circEGFR not only suppresses tumor growth but also enhances sensitivity to pirarubicin, offering a potential approach to overcoming chemotherapy resistance in TNBC patients. This finding underscores the therapeutic potential of targeting circEGFR in combination with chemotherapy to improve treatment efficacy. Further exploration of the circEGFR/miR‐1299/EGFR pathway is essential to deepen our understanding of TNBC's molecular dynamics and to develop targeted therapies aimed at mitigating chemoresistance and limiting tumor progression [59].

### 7.7 Hsa_circ_000284

Hsa_circ_000284 is significantly overexpressed in BC tissues and plasma, with its elevated levels correlating with advanced disease stages and lymph node involvement—key indicators of poor prognosis. Its detectability in plasma highlights its potential as a non‐invasive biomarker for BC diagnosis and monitoring, providing a tool for tracking disease progression associated with clinical parameters [60].

The overexpression of circRNA_000284 in advanced breast cancer is closely associated with factors such as lymph node involvement, which indicates a greater likelihood of metastasis and poorer clinical outcomes. This association underscores its potential use in evaluating disease severity and prognosis in patients [60].

CircRNA_000284 exerts its effects by acting as a sponge for miR‐506, reducing the regulatory impact of miR‐506 on its target mRNAs. One of the critical targets of miR‐506 is SNAIL2, a transcription factor known to drive epithelial‐mesenchymal transition (EMT), a fundamental process in cancer metastasis. Through the circRNA_000284/miR‐506/SNAIL2 axis, circRNA_000284 upregulates SNAIL2 expression, thereby promoting EMT and enhancing metastatic potential in BC [60]. This pathway highlights the role of circRNA_000284 in facilitating cancer progression through modulation of EMT signaling, positioning it as a significant factor in BC pathogenesis.

### 7.8 Hsa_circ_0042881

Hsa_circ_0042881 is significantly upregulated in breast tumor tissues compared to adjacent non‐tumor tissues, and elevated levels of this circRNA are also detected in the plasma of breast cancer patients. Its overexpression is associated with advanced TNM stages and larger tumor sizes, suggesting its potential as a marker for more aggressive disease. The diagnostic value of hsa_circ_0042881 is supported by an area under the curve (AUC) of 0.802, highlighting its utility as a non‐invasive biomarker for BC diagnosis associated with clinical parameters [61].

The high expression levels of circ_0042881 in both breast tumor tissues and plasma correlate with clinical indicators of advanced disease, such as larger tumor size and higher TNM stages. This association reinforces its role as a potential biomarker for evaluating disease progression and severity in breast cancer patients [61].

Circ_0042881 contributes to BC pathogenesis through its role as a miRNA sponge for miR‐217, which regulates the mRNA of son of sevenless 1 (SOS1). By sequestering miR‐217, circ_0042881 releases SOS1 from miRNA‐mediated inhibition, leading to the activation of the RAS signaling pathway. This activation subsequently stimulates downstream MEK/ERK and PI3K/AKT pathways, which are critical for promoting cell proliferation, migration, invasion, and metastasis. The circ_0042881/miR‐217/SOS1 axis thus represents a key oncogenic signaling pathway in BC, facilitating tumor growth and metastatic behavior [61]. Given the involvement of circ_0042881 in activating multiple oncogenic pathways, targeting the circ_0042881/miR‐217/SOS1 axis offers a promising therapeutic strategy.

## 8 Downregulated Plasma circRNAs in BC Patients

### 8.1 Hsa_circ_0068033

Hsa_circ_0068033 has been identified as significantly downregulated in the plasma of BC patients, contrasting with several upregulated circRNAs that often act as oncogenes. Microarray analysis followed by RT‐PCR validation confirmed lower levels of hsa_circ_0068033 in BC patients, indicating its potential role as a tumor suppressor [17]. This finding aligns with previous studies showing decreased expression of hsa_circ_0068033 in BC tissues compared to normal tissues, with a diagnostic area under the curve (AUC) of 0.84, supporting its utility as a non‐invasive biomarker for BC detection [62].

The expression of hsa_circ_0068033 is inversely correlated with BC progression, showing significant associations with advanced TNM stages (*p* = 0.023) and larger tumor sizes (*p* = 0.021). These correlations highlight hsa_circ_0068033 as a potential biomarker for assessing disease severity and prognosis [62].

Functionally, hsa_circ_0068033 exerts tumor‐suppressive effects by sponging miR‐659. This miRNA interaction reduces the availability of miR‐659 to regulate downstream target genes involved in oncogenic pathways, thereby inhibiting critical processes such as cell proliferation, migration, invasion, and colony formation in BC cells. Additionally, hsa_circ_0068033 has been shown to induce cell death, suggesting its role in activating apoptotic pathways. Through the hsa_circ_0068033/miR‐659 axis, this circRNA regulates pathways that suppress tumor progression, positioning it as a crucial modulator in BC pathogenesis [62]. This sponging action not only underscores the circRNA's regulatory capacity but also its potential therapeutic implications in targeting specific pathways involved in cancer progression.

### 8.2 Hsa_circ_0104824

Recent research has identified hsa_circ_0104824 as significantly downregulated in both BC tissues and plasma, with AUC values of 0.82 and 0.84, respectively [63]. These findings suggest that hsa_circ_0104824 could serve as a valuable non‐invasive biomarker for the diagnosis and monitoring of BC progression associated with clinical parameters.

The decreased expression levels of hsa_circ_0104824 in BC tissue and plasma samples are associated with disease presence and may reflect tumor burden, supporting its potential as a diagnostic and prognostic marker. The high AUC values in both tissue and plasma further underscore its reliability for early detection and disease monitoring [63].

Hsa_circ_0104824 appears to influence BC progression by acting as a miRNA sponge. Functional analyses revealed that it can target multiple miRNAs, including miR‐1278, miR‐140‐3p, miR‐197, miR‐599, and miR‐637, as identified through circMIR and TargetScan analyses. By sequestering these miRNAs, hsa_circ_0104824 may indirectly regulate downstream signaling pathways involved in cell proliferation and differentiation. This sponging activity suggests a potential regulatory role in preventing malignant transformation, though the specific pathways impacted by these miRNAs in BC still require further elucidation [63]. However, the molecular basis of hsa_circ_0104824 in BC metastasis and tumorigenesis requires further investigation.

### 8.3 Hsa_circ_FOXO3

Hsa_circ_FOXO3 is significantly downregulated in TNBC and is associated with more aggressive disease characteristics, including lymph node metastasis and poorer patient outcomes. This trend suggests that circ‐FOXO3 plays a role in TNBC progression and highlights its potential as a biomarker for assessing disease aggressiveness and predicting patient prognosis. Higher levels of circ‐FOXO3 are linked to less aggressive TNBC features and more favorable prognostic outcomes, indicating its utility in predicting disease progression and its potential as a therapeutic target associated with clinical parameters [64].

The decreased expression of circ‐FOXO3 in TNBC correlates with indicators of advanced disease, such as lymph node metastasis, underscoring its relevance as a prognostic marker. Its association with less aggressive clinical features in TNBC patients with higher circ‐FOXO3 levels supports its role in evaluating disease severity and likely outcomes [64].

At the molecular level, circ‐FOXO3 acts as a tumor suppressor by interacting with Wolf‐Hirschhorn syndrome candidate 1 (WHSC1). This interaction inhibits WHSC's nuclear localization, thereby blocking its ability to mediate the methylation of histone H3 at lysine 36 (H3K36me2) at the Zeb2 promoter. Zeb2 is a critical regulator of epithelial‐mesenchymal transition (EMT), a process essential for cancer metastasis. By preventing WHSC1 from activating Zeb2, circ‐FOXO3 effectively inhibits EMT, thereby reducing TNBC growth and metastatic potential. This circ‐FOXO3/WHSC1/Zeb2 axis underscores the role of circ‐FOXO3 in regulating key signaling pathways that drive TNBC progression [64]. Further research into therapeutic approaches that target this pathway may lead to improved treatment outcomes for TNBC patients, offering a novel avenue for addressing this aggressive cancer subtype.

## 9 Challenges, Limitations, and Controversies in circRNA Research

Despite the promising potential of circRNAs as biomarkers [83, 84] and therapeutic targets in BC, several challenges and controversies hinder their clinical translation. Reproducibility remains a major issue, as differences in sample preparation, sequencing techniques, and bioinformatics tools often result in inconsistent findings across studies. Additionally, tumor heterogeneity and variations in circRNA expression between tissue and plasma samples complicate the identification of universal biomarkers. Also, the high cost and complexity of detection methods, such as RT‐ddPCR and RNA‐Sequencing, limit their widespread clinical application. Functional ambiguities also persist, with debates surrounding whether circRNAs primarily act as passive byproducts of splicing or active regulators through mechanisms like miRNA sponging and protein‐coding potential. Moreover, a lack of standardized protocols and large‐scale longitudinal studies hampers their validation as reliable diagnostic or prognostic tools. Addressing these limitations requires multi‐center collaborations, the development of more cost‐effective and sensitive detection technologies, and further research to clarify their functional roles and establish robust clinical workflows.

## 10 Conclusion

Our review underscores the significant role of circulating circRNAs (ccircRNAs) as potential biomarkers in BC. The dysregulation of specific circRNAs in plasma provides insights into their mechanisms, particularly as miRNA sponges that modulate critical oncogenic and tumor‐suppressive pathways. These interactions highlight the theoretical importance of circRNAs in breast cancer pathogenesis by regulating gene expression, cell proliferation, migration, and metastasis. Such a regulatory network suggests a new layer of complexity in breast cancer biology and positions circRNAs as valuable targets for in‐depth research.

From a translational perspective, the distinct stability and detectability of ccircRNAs in blood make them promising candidates for non‐invasive liquid biopsies in breast cancer. Their diagnostic value, demonstrated by the high AUCs of certain circRNAs like hsa_circ_0001785 and hsa_circ_0108942, underlines their utility in early detection and monitoring of breast cancer. Furthermore, the association of these circRNAs with advanced disease stages or metastasis supports their prognostic potential, allowing clinicians to better stratify patient risk and customize treatment plans.

Beyond diagnosis, circRNAs offer potential therapeutic applications. Targeting dysregulated circRNAs could disrupt pathogenic pathways in cancer cells, opening avenues for innovative therapies. As research progresses, circRNA‐based therapies, such as using circRNA mimics or inhibitors, could provide a novel approach to regulating cancer progression and overcoming treatment resistance.

In conclusion, while more studies are required to fully elucidate the biological roles of these circRNAs, our findings lay a foundation for their integration into clinical settings. The dual potential of circRNAs as both diagnostic and therapeutic tools highlights a promising direction for personalized medicine in breast cancer care. This review emphasizes the need to bridge foundational research with clinical applications to improve patient outcomes and advance breast cancer management.

## Author Contributions

S.A. investigated, drafted the manuscript, and created the figures; G.A.‐T. supported the investigation and contributed to writing the original draft; A.S.N.L. contributed to the investigation and writing the original draft; A.R.M. contributed to the investigation and writing the original draft; P.I. conceptualized the main idea and supervised the manuscript by reviewing and editing the draft and validation. All the authors have read and approved the final manuscript.

## Conflicts of Interest

The authors declare no conflicts of interest.

## Data Availability

Data sharing not applicable to this article as no datasets were generated or analysed during the current study.
